# Incidence of Tuberculosis and the Predictive Value of ELISPOT and Mantoux Tests in Gambian Case Contacts

**DOI:** 10.1371/journal.pone.0001379

**Published:** 2008-01-02

**Authors:** Philip C. Hill, Dolly J. Jackson-Sillah, Annette Fox, Roger H. Brookes, Bouke C. de Jong, Moses D. Lugos, Ifedayo M. Adetifa, Simon A. Donkor, Alex M. Aiken, Stephen R. Howie, Tumani Corrah, Keith P. McAdam, Richard A. Adegbola

**Affiliations:** Bacterial Diseases Programme, Medical Research Council (MRC) Laboratories, Banjul, The Gambia; University of Cape Town, South Africa

## Abstract

**Background:**

Studies of Tuberculosis (TB) case contacts are increasingly being utilised for understanding the relationship between *M. tuberculosis* and the human host and for assessing new interventions and diagnostic tests. We aimed to identify the incidence rate of new TB cases among TB contacts and to relate this to their initial Mantoux and ELISPOT test results.

**Methods and Findings:**

After initial Mantoux and ELISPOT tests and exclusion of co-prevalent TB cases, we followed 2348 household contacts of sputum smear positive TB cases. We visited them at 3 months, 6 months, 12 months, 18 months and 24 months, and investigated those with symptoms consistent with TB. Those who were diagnosed separately at a government clinic had a chest x-ray. Twenty six contacts were diagnosed with definite TB over 4312 person years of follow-up (Incidence rate 603/100,000 person years; 95% Confidence Interval, 370–830). Nine index and secondary case pairs had cultured isolates available for genotyping. Of these, 6 pairs were concordant and 3 were discordant. 2.5% of non-progressors were HIV positive compared to 12% of progressors (HR 6.2; 95% CI 1.7–22.5; p = 0.010). 25 secondary cases had initial Mantoux results, 14 (56%) were positive ; 21 had initial ELISPOT results, 11 (52%) were positive; 15 (71%) of 21 tested were positive by one or the other test. Of the 6 contacts who had concordant isolates with their respective index case, 4 (67%) were Mantoux positive at recruitment, 3 (50%) were ELISPOT positive; 5 (83%) were positive by one or other of the two tests. ELISPOT positive contacts, and those with discordant results, had a similar rate of progression to those who were Mantoux positive. Those negative on either or both tests had the lowest rate of progression.

**Conclusions:**

The incidence rate of TB disease in Gambian TB case contacts, after screening for co-prevalent cases, was 603/100,000 person years. Since initial ELISPOT test and Mantoux tests were each positive in only just over half of cases, but 71% were positive by one or other test, positivity by either might be the best indication for preventive treatment. These data do not support the replacement of the Mantoux test by an ELISPOT test in The Gambia or similar settings.

## Introduction

Household contacts of tuberculosis (TB) cases have a relatively high prevalence of TB disease when screened [1] and a higher incidence rate of TB disease than the general population in the first years after exposure, the rate varying across different locations and populations[2]–[5]. It is likely that contacts of known TB cases contribute greatly to the overall TB disease burden in certain settings. For example, in West Africa, up to 45% of newly diagnosed TB cases have a known TB contact [6], [7]. TB case contact studies are being engaged increasingly as platforms for studying TB and have potential in relation to the assessment of new diagnostic tests and interventions, including vaccines. However, more needs to be known about the epidemiology of TB in case contacts to assist in the planning and implementation of such studies.

Cross-sectional studies have suggested promise for interferon-gamma T cell based tests in the diagnosis *M. tuberculosis* infection and TB disease[8]–[12]. However, longitudinal studies suggest caution [13], [14], especially in interpreting a result when there is no known recent TB contact[15]. Furthermore, it is not known what proportion of those who are TB case contacts, and become cases themselves, have a positive test from an initial screen. Recently, the British National Institute for Health and Clinical Excellence (NICE) published recommendations suggesting that the traditional Mantoux skin test and an interferon-gamma test be used in a two-step manner – the Mantoux test as a screening tool and an interferon-gamma test as confirmation [16]. The evidence to support such an approach is lacking.

In this study we followed a large number of TB case contacts for the development of TB disease and related their initial Mantoux and ELISPOT test results to the likelihood of progression to TB disease.

## Materials and Methods

### Study population

Between June 2002 and October 2004, TB cases and their household contacts were enrolled in a prospective cohort study in the Greater Banjul region of The Gambia, West Africa. We recruited, from the major government TB clinic and the Medical Research Council (MRC) outpatients' clinic, sputum smear positive pulmonary TB cases aged at least 15 years old, who had at least one household contact living with them. An index case was defined as the first TB case identified in a household. Household contacts were defined as individuals at least 6 months of age living the majority of the time on the same compound as the respective index TB case, sharing meals and identifying a common household head. Written informed consent was obtained from all adults in the study and parents/guardians of children under 15 years of age. The study was approved by the combined Gambia Government/MRC National Ethics Committee of The Gambia.

At the time of recruitment, contacts were interviewed and immediately afterwards had a blood sample taken for ELISPOT and HIV test, followed by a Mantoux test (2 Tuberculin Units [TU], PPD RT23 Statens Serum Institut, http:/www.ssi.dk). A maximum of 12 contacts had an ELISPOT test per day, others were randomly excluded. Those with symptoms, or a positive Mantoux test, were investigated for TB. The current policy in The Gambia is not to give Mantoux positive individuals preventive therapy. A co-prevalent TB case was defined as a household contact diagnosed with TB who had symptoms that commenced within 2 months of the recruitment of the respective index case. The results of the initial screening, yielding 33 co-prevalent cases, have been published elsewhere[1]. We performed five follow-up visits (at 3, 6, 12, 18 and 24 months after recruitment) to each household and referred those with symptoms to the MRC TB clinic. All study participants had free access to the MRC TB clinic for treatment for any illness during this period. Any patients with symptoms of pulmonary disease received a chest X-ray and sputum analysis for AFB smear analysis and culture. If TB disease was bacteriologically confirmed, or clinically suspected in smear-negative or extra-pulmonary cases, patients were referred for a free standard six month TB treatment course to the Gambian National TB Treatment program. For those contacts who died, we designed a modified verbal autopsy form, focused on TB symptoms. This was administered by a trained field worker to family members and interpreted by clinicians.

### Secondary Case definition

All contacts with symptoms, consistent with TB, that commenced at least 2 months after their respective index case was diagnossed, or with a positive Mantoux test (at least 10 mm of induration) at any follow-up time point, were considered to be possible secondary TB cases. They were asked to have a chest X-ray and provide sputum if they had a productive cough. All those initially Mantoux negative at recruitment were asked to have a repeat Mantoux test at 3 months. Those that were Mantoux positive were offered a chest X-ray. A diagnosis of a secondary TB case was based on the chest X-ray and sputum smear and culture results, and/or their response to a course of TB treatment if a trial of therapy had been considered appropriate. They were classified as having pulmonary or extra-pulmonary disease by consensus of two infectious diseases physicians and one paediatrician specialised in the diagnosis and management of TB cases.

An alternative strategy was devised with respect to those contacts who sought a diagnosis only at a government clinic, where sputum culture was not available. Names and ages of all TB cases treated at the government health clinics during the course of the study were recorded. Those that matched with contacts participating in our study, using an age category matching within 5 years, were re-visited to confirm whether they had had TB treatment or not. Those that confirmed having taken treatment, were asked to have a chest X-ray. Each radiograph was reviewed by the two infectious diseases physicians, and/or pediatrician when the participant was a child. After review, a consensus opinion regarding the diagnosis of a secondary TB case was formed . Those with no evidence of TB on chest X-ray were defined as ‘not definite’.

### Laboratory procedures

Sputum samples were prepared for smear using auramine-phenol, and cultured using Lowenstein-Jensen medium and the BACTEC 9000 MB system (Becton Dickinson) for identification of *M. tuberculosis*, as previously described[17]. For molecular subtyping of index case isolates, we extracted mycobacterial DNA using CTAB and chloroform[18]. We performed spoligotyping using membranes from Isogen Biosciences (http:/www.isogen-lifescience.com), as previously described[19]. When the isolate of an index case and the isolate of a diseased contact from the same household shared the same spoligotype pattern (genotype), these were referred to as concordant genotypes. Isolates that shared the same spoligotype pattern except for one of 43 spacers, were also classified as concordant genotypes. All others were classified as discordant genotypes.

The *ex-vivo* ELISPOT assays for IFN-γ were performed on fresh samples onsite as previously described.[11] Pooled sequential 15-mer peptides, overlapping by 10 amino-acid residues, of ESAT-6 and CFP-10 proteins (ABC, Imperial College, London, UK) were used as stimulatory antigens at 5 µg/ml. The positive control was phytohaemaglutinin (PHA; Sigma-Aldrich, UK). All antigens were tested in duplicate wells. Assays were scored by an ELISPOT counter (AID-GmbH, Strassberg, Germany). Positive test wells were pre-defined as containing at least eight spot forming units (SFU) more than negative control wells.[20] For a positive ESAT-6/CFP-10 result it was necessary for at least one of the pools of overlapping peptides to be positive. PHA wells were set to at least 150 SFU/well/2×10^5^ above negative control wells. Negative control wells were required to have less than 20 SFU. These criteria are the same as we have previously documented and are more stringent than those recommended for the commercial T-spot assay[21].

Testing for HIV-1 and HIV-2 infection was by competitive ELISA (Wellcome Laboratories, Dartford, Kent, Uk) and Western blot (Diagnostics Pasteur, http:/www.sanofipasteur.com) as previously described[22]. Those individuals who tested positive were referred to the onsite MRC HIV clinic for clinical care and follow-up, being eligible for consideration for free anti-retroviral therapy according to set criteria.

### Data management and statistical analysis

The number of SFU in each ELISPOT well were automatically entered into a database. All other data were entered using double data entry into an ACCESS database and checked for errors[23]. We used Cox proportional hazards regression to assess the relationship between risk factors and rate of progression to disease in TB contacts, having confirmed non-violation of the proportional hazards assumption. Results were reported as unadjusted and adjusted hazard ratios (HR) and their 95% confidence intervals (CI). All statistical analyses were conducted using Stata software (version 8; Stata Corp, http://www.statacom).

## Results

**Figure 1:**
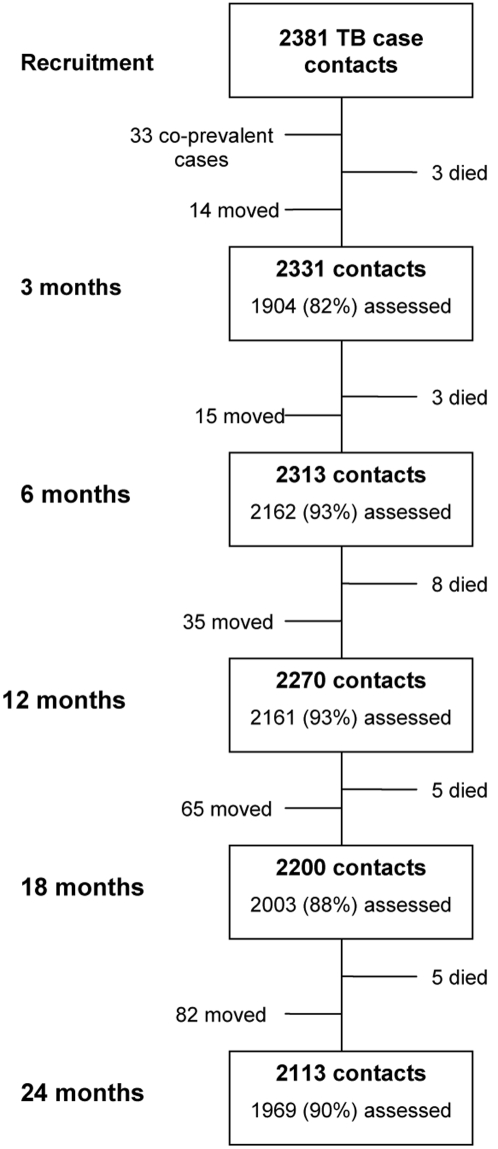
Study profile.

Of the 2381 contacts of 317 sputum smear and culture positive TB cases, 33 were excluded from follow-up, having been identified as co-prevalent cases. Figure 1 shows the numbers of contacts assessed at each of the five follow-up points over 2 years: 24 died and 191 were lost to the study through migration out of the study area; 82–93% of those resident at each time point were assessed for symptoms of TB. Twenty six contacts were diagnosed with as secondary TB cases over 4312 person years of follow-up. A further 6 contacts were treated for TB but the diagnosis was considered not definite. Of the 24 who died duriing follow-up: four were HIV positive, two of whom had been a TB suspect but had normal chest X-rays; 11 clearly died of a non-TB cause; five had some history on verbal autopsy suspicious for TB, four of these had been investigated during follow-up and had normal investigations; and a verbal autopsy was not possible for four.

**Figure 2:**
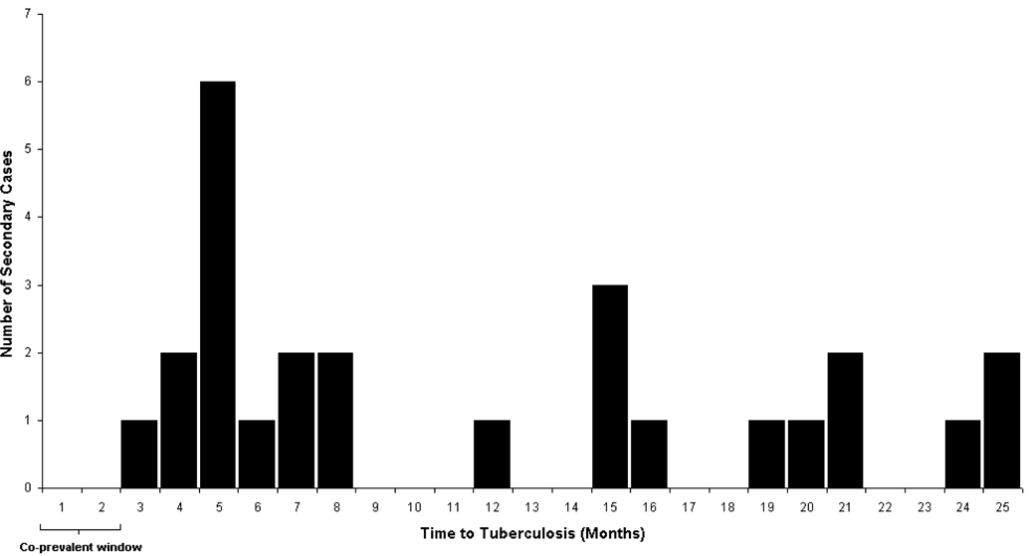
Timing of presentation for diagnosis of incident TB cases after exposure to their respective index case.

Figure 2 shows the period from enrolment to the time when the contact first became a TB suspect: 14 were diagnosed in the first 6 months after the co-prevalent window period, the other 12 were distributed over the next 17 months. Table 1 shows the characteristics of those who progressed to disease versus those who did not. Progressors had a similar gender and ethnicity split than those who did not progress. Interestingly there was not a strong relationship with sleeping proximity to a TB case. As expected, while only 2.5% of non-progressors were HIV positive, 12% (3 of 24) progressors were HIV positive at recruitment. This difference was significant in the univariable (HR 6.0; 95% CI 1.8–20.0; p = 0.020) and multivariable analysis (HR 6.2; 95% CI 1.7–22.5; p = 0.010), and accounted for some of the age difference between progressors and non-progressors. No information was available regarding the level of immunosuppression of the HIV positive progressors. BCG scar was not significantly associated with protection against progression to disease.

**Table 1:** Baseline information of TB case contacts who progressed to disease (n = 26) and those who did not (n = 2322).

Characteristic	Progressors (n = 26)	Non-progessors (n = 2322)	HR (95% CI)	*p-*value	Adj HR (95% CI)	Adj *p-*value
Age, years						
0.5–10	7 (26.9)	861 (37.1)	1.0		1.0	
11–25	10 (38.5)	891 (38.4)	1.4 (0.5–3.6)		1.1 (0.4–2.9)	
>25	9 (34.6)	590 (25.4)	1.9 (0.7–5.0)	0.46	1.2 (0.4–3.4)	0.95
Mean (STD)	24.8 (17.6)	18.6 (15.5)				
Median (range)	21.5 (2–70; IQR 10–27)	15.5 (0.5–100; IQR 7–25)				
Sex						
Female	15 (57.7)	1236 (53.2)	1.0			
Male	11 (42.3)	1086 (46.8)	0.8 (0.4–1.7)	0.55		
Ethnic group						
Mandinka	5 (19.2)	749 (32.3)	1.0		1.0	
Wolof	6 (23.1)	305 (13.1)	2.7 (0.9–8.0)		3.4 (1,1–11.0)	
Fula	2 (7.7)	198 (8.5)	1.5 (0.3–4.3)		1.7 (0.3–9.3)	
Jola	10 (38.5)	614 (26.4)	3.4 (1.0–11.3)		5.1 (1.4–18.5)	
Other	3 (11.5)	456 (19.6)	1.0 (0.2–4.3)	0.16	1.4 (0.3–6.1)	0.085
Sleeping proximity to a TB case						
Different house	6 (23.1)	727 (31.0)	1.0			
Different room	14 (53.8)	1092 (46.6)	1.5 (0.6–4.0)			
Same room	6 (23.1)	523 (22.3)	1.5 (0.5–4.5)	0.68		
HIV status (n = 2009)						
Negative	22 (88)	1938 (97.7)	1.0		1.0	
Positive	3 (12)	46 (2.3)	6.0 (1.8–20.0)	0.020	6.2 (1.7–22.5)	0.010
BCG scar status						
Absent/uncertain	18 (69.2)	1349 (58.1)	1.0		1.0	
Present	8 (30.8)	973 (41.9)	0.6 (0.3–1.4)	0.25	0.7 (0.3–1.8)	0.51

Data are n(%), unless otherwise stated.

Table 2 provides details of each individual TB secondary case in relation to their recruitment Mantoux and ELISPOT results. At recruitment, 14 (56%) of 25 that were tested, and later diagnosed as cases, were Mantoux positive; 11 (52%) of 21 tested were ELISPOT positive; and 15 (71%) of 21 tested were positive by one or the other of the two tests. The agreement, in the secondary cases, between the two tests at recruitment was 61.9% (kappa = 0.24). Nine index case and secondary case pairs had cultured isolates available for molecular genotyping. Of these, 6 pairs were concordant and 3 were discordant. Of the 6 contacts who had concordant isolates with their respective index case, 4 (67%) were Mantoux positive at recruitment, 3 (50%) were ELISPOT positive and 5 (83%) were positive by one or other of the two tests.

**Table 2:** Characteristics and type of TB in secondary cases, their recruitment Mantoux and ELISPOT results and details of the molecular genotyping results of index case and secondary case isolates if available.

Age (yrs)	Sex	BCG scar	Type of TB*	Time to illness (months)	HIV status	Sleep prox	Mantoux (mm)	Mantoux (pos/neg)	ELISPOT ESAT-6 (spots/2×10^5^)	ELISPOT CFP-10 (spots/2×10^5^)	ELISPOT (pos/neg)	Same isolate
2	F	No	EPTB	4.7	Neg	Diff house	0	Neg	3	0	Neg	-
6	F	Unsure	EPTB	4.4	Neg	Diff room	0	Neg	0	17	Pos	-
6	M	No	EPTB	7.7	Neg	Same room	30	Pos	111	12	Pos	-
7	F	Yes	SPXRPTB	4.7	Neg	Same room	ND	ND	ND	ND	ND	-
8	F	No	SPCPTB	6.3	Neg	Diff room	17.5	Pos	ND	ND	ND	No
10	F	No	SNXRPTB	3.6	Neg	Diff house	5	Neg	58	2	Pos	-
10	M	Yes	SNCPTB	5.6	Neg	Same room	0	Neg	0	0	Neg	Yes
14	M	No	SPXRPTB	6.9	Neg	Diff room	23.5	Pos	34	6	Pos	-
16	F	Yes	SPCPTB	24.9	Neg	Diff room	21.5	Pos	92	19	Pos	Yes
18	F	Unsure	SPXRPTB	4.9	Neg	Diff room	0	Neg	1	1	Neg	-
19	F	Yes	SNCPTB	20.9	Neg	Diff room	18	Pos	7	3	Neg	Yes
21	M	No	SNCPTB	3.1	Neg	Diff room	15.5	Pos	ND	ND	ND	-
21	M	Yes	SPXRPTB	15.6	Neg	Same room	22.5	Pos	1	0	Neg	-
22	F	Yes	SPXRPTB	11.2	Neg	Diff room	14	Pos	8	0	Pos	-
22	M	Unsure	SPCPTB	14.5	Neg	Diff room	13	Pos	0	0	Neg	Yes
23	M	No	SPCPTB	2.1	Neg	Diff house	17	Pos	92	117	Pos	No
25	F	No	SPXRPTB	14.7	ND	Diff room	16	Pos	ND	ND	ND	-
26	M	Unsure	SPCPTB	18.7	Neg	Same room	20	Pos	61	8	Pos	Yes
27	F	No	SPXRPTB	7.3	Pos	Diff room	0	Neg	ND	ND	ND	-
27	M	Unsure	SPCPTB	24.8	Neg	Diff room	0	Neg	3	9	Pos	Yes
40	M	No	SPXRPTB	4.3	Pos	Same room	0	Neg	2	4	Neg	-
45	M	Yes	SPXRPTB	14.6	Neg	Diff room	0	Neg	0	0	Neg	-
46	F	No	SPXRPTB	23.9	Pos	Diff house	20	Pos	185	26	Pos	-
53	F	No	SPXRPTB	4.8	Neg	Diff room	15.5	Pos	2	0	Neg	-
60	F	Unsure	SNCNXRPTB	20.4	Neg	Diff house	0	Neg	14	17	Pos	-
70	F	Yes	SNCPTB	19.7	Neg	Diff house	0	Neg	1	3	Neg	No

* Abbreviations used: EP, extrapulmonary; SPXRP, sputum positive/X-ray positive; SNXRP, sputum negative/X-ray positive; SNCP, sputum negative/culture positive; SPCP, sputum positive/culture positive; SNCNXRP, sputum negative/culture negative/X-ray positive

Table 3 shows the incidence rate of progression to disease according to recruitment Mantoux and ELISPOT results. Those contacts who were ELISPOT positive and those who were ELISPOT negative at recruitment had a similar rate of progression to disease as those who were Mantoux postive and negative respectively. Those negative on either or both tests had the lowest incidence rate of progression. Of note, those with discordant results had a similar rate of progression to those who were positive on either test. Taking into consideration the 21 cases with ELISPOT and Mantoux results at recruitment in relation to current international guidelines for preventive therapy: 15 would have been captured by accepting a positive result on one or both tests, 11 would have been captured by either the Mantoux or ELISPOT test, but only 7 would have been captured by a requirement to be positive on both tests–over half (8 of 15) that were intially positve by either test had discordant results at recruitment. Using Cox regression modelling, the crude and adjusted Hazard ratios for a positive test in relation to becoming a secondary case were: 1.9 (95% CI: 1.0–4.7) and 1.8 (95% CI 0.8–4.1) respectively for the Mantoux test and 1.9 (0.8–4.5) and 1.8 (0.8–4.2) for the ELISPOT test.

**Table 3:** Incidence rate of progression to disease, in TB case contacts, according to initial Mantoux and/or ELISPOT results.

Mantoux/ELISPOT result	Progressors/population	Incidence/100,000 person years (95% confidence interval)
All	26/2348 (1.1%)	603 (370–830)
Mantoux +	14^a^/843 (1.7%)	902 (430–1370)
Mantoux −	11^a^/1387 (0.8%)	431 (180–680)
ELISPOT +	11^b^/649 (1.7%)	924 (380–1460)
ELISPOT −	10^b^/1087 (0.9%)	502 (190–810)
Mantoux +/ELISPOT +	7^c^/428 (1.6%)	886 (240–1540)
Mantoux + or ELISPOT +	15^c^/835(1.8)	980 (570–1390)
Mantoux +/ELISPOT −	4^c^/230 (1.7%)	959 (20–1900)
Mantoux −/ELISPOT +	4^c^/177 (2.3%)	1242 (30–2450)
Mantoux −/ELISPOT −	6^c^/813 (0.7%)	400 (80–720)

a 25 cases and 2205 non-progressors had a Mantoux test;

b 21 cases and 1715 non-progressors had an ELISPOT done;

c 21 cases and 1627 non-progressors had both a Mantoux and ELISPOT test done.

## Discussion

After an initial screen for co-prevalent disease[1], we actively followed over 2000 household contacts of sputum smear positive TB cases for a total of 4312 person years. Twenty-six secondary cases were identified, giving an incidence rate of 603/100,000 person years. HIV positivity, despite a low prevalence in this population, was the one significant risk factor for progression to TB disease. We were able to calculate the incidence rates of secondary disease in relation to initial Mantoux and ELISPOT results and to show how many secondary cases were ‘captured’ by a positive test. Only 15 (71%) of the 21 secondary cases with recruitment results were positive on either test. Furthermore 4 Mantoux positive secondary cases were ELISPOT negative and 4 ELISPOT positive secondary cases were Mantoux negative. The results of this study have implications for the design of trials of new interventions, particularly vaccines, in Africa, and the revision of current guidelines for TB control.

The 33 (1.5%) co-prevalent cases and the 26 secondary cases identified in this study population can be compared with the number identified in other TB case contact studies, although few have presented incidence rates of secondary disease - which can only be calculated when the total person time of follow-up is known. Guwatudde et al [2] conducted a case contact study in Uganda, following 1206 contacts of 423 TB cases for 2 years. As in our study, over half of the TB cases in these African TB contacts were found at an initial screen - they identified 51 (4.2%) co-prevalent cases and 25 secondary cases. The median age of the secondary cases was 31 years and 13 (52%) were HIV positive. The higher rate of HIV positivity compared to our study explains a large part of the higher prevalence of cases at screening and the larger relative number of secondary cases at follow-up. Furthermore, a higher proportion (66% vs 38%) of the Ugandan contacts were Mantoux positive at recruitment, suggesting a higher background rate of *M. tuberculosis* infection. Egsmose et al [3], as part of a study in rural Kenya, found that 17 (4.3%) of 392 TB case contacts became ‘tubercle bacilli excretor’ TB cases within one year, after exclusion of those diagnosed with TB before enrolment. Puffer et al [4] followed 1358 household contacts of 298 ‘sputum positive’ American TB cases in the 1940s for 8754 person years and identified 30 cases of clinical TB: the overall attack rate of clinical TB was 690/100,000 person years. Ferebee et al [5] found 479 (1.9%) cases of TB in 25,512 American household contacts of TB cases (not limited to sputum smear positive cases) in the 1950s. Of the remaining contacts 12,594 were assigned not to receive isoniazid: in this group, 107 (0.8%) secondary cases were identified over one year of follow-up; 62 of these had pulmonary disease.

It is possible that we have underestimated the true number of secondary cases in this study. Some of those who died may have had TB, although this appeared unlikely in most from the verbal autopsies. We also excluded some of those who had undergone TB treatment, because they did not meet our criteria for diagnosis. Some of these individuals may well have had TB disease. We have also shown that some cases preferentially access the government clinic for diagnosis in The Gambia, even with free access to the MRC study clinic. While we are confident that we have captured those that were diagnosed at the government clinic, we suspect strongly that stigma is a significant impediment to the acknowledgement of both symptoms and to seeking a TB diagnosis and treatment in The Gambia and many other African settings. Furthermore, as identified by Guwatudde et al[2], the limitations of current diagnostic tools make an underestimation of the true number of secondary cases possible, especially in relation to the difficult diagnosis of TB in children. Under-diagnosis of secondary TB in children would lead to a selection bias, if present, and has implications for the interpretation of age as a predictor of progression to disease.

This study sheds new light on the appropriateness of major TB guidelines, in particular the NICE guidelines – the results do not support the two-step appraoch that is advocated,[16] at least in settings such as The Gambia. Using such an approach, only seven of the 21 progressors in our study, that were initially tested by both tests, would have been eligible for preventive treatment. This compares to 15 who were positive on one or other test. These results were consistent with those obtained when considering only the initial results of those six contacts with an isolate concordant by genotyping with that of their respective index case. Few data from other studies are available to shed more light on this issue. In a retrospective study of practice in 120 child TB suspects in Newcastle, Taylor et al[24] identified 5 TB cases: 4 had a positive Mantoux test at the beginning of the investigations, 3 had a positive Quantiferon T cell assay test. This, together with the results presented here, suggest caution in the application of the NICE guidelines.

One might consider, as an alternative strategy, preventive treatment for those who are positive on either test. A possible approach would be to treat all those who are Mantoux positive, then perform a T cell based test on those who are Mantoux negative, treating those with a positive result. Thus, instead of testing all Mantoux positive individuals with a T cell assay as is recommended, one would test all Mantoux negative individuals. These issues can be considered in terms of numbers needed to treat to prevent one TB case. From our results, 60 Mantoux positive contacts need to be treated to prevent a case, compared to 59 ELISPOT positive contacts and 90 contacts overall (ie. regardless of test result). Taking those positive by skin test plus those skin test negative contacts that are ELISPOT positve, 56 would need to be treated to prevent one TB case. These calculations assume 100% efficacy of prophylactic treatment, which would be expected to be less than 90% in reality [25].

It would be important to consider repeating each test in those initially negative as we have previously shown that some who later become cases, converted to a positive test 3 months after the initial screen [15]. Furthermore, it is of note that even those TB case contacts that were both Mantoux and ELISPOT negative had an incidence rate of secondary disease of 400/100,000 person years. This compares to the incidence rate of all cases of TB of 242/100,000 per year for the whole Gambian population (http://www.who.int/globalatlas/predefinedreports/tb/PDF_Files/gmb.pdf), regardless of Mantoux and/or ELISPOT status prior to progression to disease. One could therefore make a case, in making policy for prophylactic treatment, for treating all those who have a known contact with a sputum smear positive TB case in The Gambia and similar settings. International guidelines presently suggest such a strategy only in Mantoux negative individuals with a particular susceptibility to develop TB disease[26]. Of relevance to The Gambia and other African countries in particular, HIV positive individuals cleary fall into this category.

The results of the molecular genotyping provide insights into the TB case contact model as a platform for TB research in The Gambia: six (67%) of the nine pairs of index cases and secondary cases with isolates available were concordant. Therefore, the other three secondary cases acquired their *M. tuberculosis* from someone other than their respective index case, most probably from outside their household. The mix of intra- and extra- household transmission likely varies according to the degree of TB endemnicity in the general population. In highly endemic suburbs of Capetown, South Africa, for example, isolates match on molecular subtyping in less than half (46%) of households with two TB patients, and the authors estimated that only 19% of *M. tuberculosis* transmission occurs within households [27]. Therefore, one should be cautious when interpreting findings from TB case contact studies, taking into account the level of community transmission outside households and molecular genotyping on index case and secondary case isolates should be performed when possible.

This results of this study have implications for research, clinical and public health practice. The incidence rate that we have documented here can form a guide for those choosing to use a TB case contact study platform for research. While approximately 5 to 6% of Mantoux or ELISPOT positive TB case contacts develop TB within 2 years in The Gambia, the majority are already cases at initial screening and it is clear that multi-site studies, with a higher overall case yield, may be needed to answer certain research questions. Such consortia are already being established. For example a Gates Grand Challenge project across multiple African sites utilises a TB case contact study model[28]. Two key conclusions can be drawn from the ELISPOT and Mantoux test results. Firstly, it seems that certain individuals respond preferentially to one or the other test, at least in the early stages of *M. tuberculosis* infection. This may be due to varying incubation periods, as we have indicated previously[15], or there may be a more fundamental immunological basis. Thus, both tests may be best used together at an initial screen – perhaps to test first with Mantoux and all those that are negative with ELISPOT-this takes advantage of the likelihood that ELISPOT is probably not subject to the booster phenomenon[29]. Secondly, where contact with an index case continues throughout treatment and conversion of both the Mantoux and ELISPOT tests occur over time[15], repeated testing should be considered. At a wider public health policy level, these results do not support replacement of the Mantoux test with a T cell based test for the diagnosis of TB infection, at least in settings such as The Gambia.
